# Extraversion Moderates the Relationship Between the Stringency of COVID-19 Protective Measures and Depressive Symptoms

**DOI:** 10.3389/fpsyg.2020.568907

**Published:** 2020-10-02

**Authors:** Indy Wijngaards, Sophie C. M. Sisouw de Zilwa, Martijn J. Burger

**Affiliations:** ^1^Erasmus Happiness Economics Research Organization, Erasmus University Rotterdam, Rotterdam, Netherlands; ^2^Erasmus School of Health and Policy Management, Erasmus University Rotterdam, Rotterdam, Netherlands; ^3^Erasmus School of Economics, Erasmus University Rotterdam, Rotterdam, Netherlands

**Keywords:** COVID-19, COVID-19 protective measures, extraversion, depressive symptoms, mental health—related quality of life

## Abstract

From the start of the COVID-19 pandemic, psychologists are theorizing that, as compared to introverts, extraverts experience more profound negative social consequences from protective measures (e.g., travel restrictions and bans on public gatherings). As the empirical evidence for this claim is lacking, this study tested the hypothesis that extraversion moderates the relationship between the stringency of COVID-19 protective measures and depressive symptoms. Our results were based on survey data from 93,125 respondents collected in the early stages of the COVID-19 pandemic (March 20–April 6, 2020) across 47 countries and publicly available data on measure stringency. Findings demonstrate that extraversion moderates the relationship between measure stringency in the early days of the pandemic and depressive symptoms. For introverts, measure stringency has a negative effect on depressive symptoms, while for extraverts, it has a positive, but non-significant effect on depressive symptoms. This study suggests that, although stringent measures generally help people to worry less and feel safer, the lifestyle associated with such measures feels more natural to introverts than to extraverts.

## Introduction

The COVID-19 pandemic has a profound negative effect on the world population’s physical and mental health (Dong et al., 2020; Van Bavel et al., 2020; World Health Organization, 2020). In varying degrees, governments all over the world imposed protective measures to contain the spread of the virus (Anderson et al., 2020; Hale et al., 2020). For instance, the Belarusian government dismissed the global coronavirus pandemic and imposed only a handful of measures. The Swedish government installed a larger number of measures but refrained from imposing a lockdown. Governments from China and Italy swiftly installed a total lockdown of the entire country.

Recent research during the early stages of the pandemic suggests that stringent measures also function as safeguard of mental health; they cause citizens to worry less and feel safer (Fetzer et al., 2020). This does not mean that protective measures bring nothing but benefits. An increasing degree of stringency of COVID-19 protective measures is typically accompanied by increased social distancing, the limiting of face-to-face contact with others by keeping space between oneself and other people outside of one’s home. Inherent by-products of social distancing are increased feelings of loneliness, frustration, worry and boredom—negative emotional states that, if left unattended, could lead to mental illness (Brooks et al., 2020; Folk et al., 2020; Galea et al., 2020).

Yet, it is unlikely that the effects of social consequences of measure stringency on mental illness are universal across all people. Drawing from pre-pandemic research, psychologists are proposing that extraversion—a personality trait characterized by sociability, assertiveness and high energy levels (John et al., 1991; Soto and John, 2017)—is one individual characteristic that could moderate the negative relationship between measure stringency and mental illness (e.g., Brogaard, 2020; Brooks and Moser, 2020; Smillie and Haslam, 2020; Steele, 2020). More specifically, they argue that there are potential advantages to being an introvert and potential disadvantages to being an extravert in countries where stringent measures are in place. The lifestyle associated with social distancing would feel more unnatural to extraverts than to introverts, as it inhibits extraverts to satisfy their strong urges to seek out social engagement (Woodcock et al., 2013), to experience pleasure and excitement (Kämpfe and Mitte, 2009), and to live in new and exciting surroundings (Oishi and Choi, 2020). Introverts, in contrast, would fare better, as the lifestyle allows them to shamelessly be alone more often and decide when and where to connect with others.

To date, however, the assumption that the social consequences of measure stringency are negative for extraverts and positive for introverts, remains largely untested. The first empirical tests based on data collected during the pandemic are inconclusive, with studies reporting negative (Płomecka et al., 2020), positive (Folk et al., 2020), or insignificant (Elmer et al., 2020; Weinstein and Nguyen, 2020) associations between extraversion and mental illness. In this study, drawing on publicly available survey data from over 90,000 respondents across 47 countries (Fetzer et al., 2020), we therefore empirically test the hypothesis that *extraversion moderates the relationship between measure stringency and depressive symptoms.* By looking at moderation effects, we aimed to further nuance Fetzer et al.’s (2020) finding that measure stringency leads to reduced depressive symptoms. We also address a general calls for research on the mental health effects of COVID-19 protective measures (Holmes et al., 2020; Van Bavel et al., 2020) and more specific calls for investigations on the interplay between personality, the experience of social distancing and mental health (Folk et al., 2020; Oosterhoff et al., 2020).

## Materials and Methods

### Participants and Procedure

**FIGURE 1:**
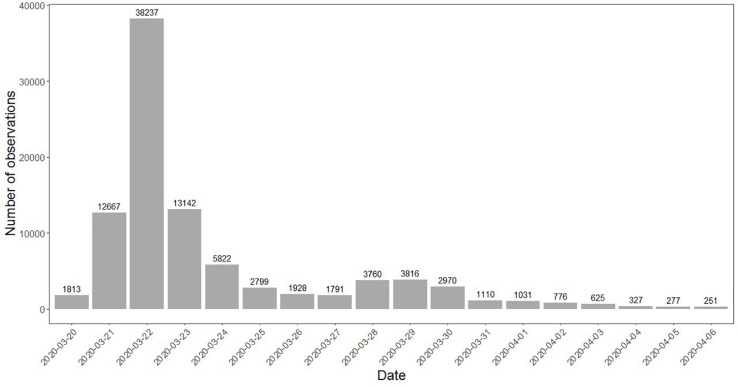
Overview of observations per day.

In this study, we utilized Fetzer et al.’s (2020) data. They used online snowball sampling to recruit respondents in the early stages of the COVID-19 pandemic (March 20–April 6, 2020), a period in which the pandemic spread rapidly, and many consequential policy decisions were made. In total, 112,136 respondents from 175 countries filled out the survey. Following recommendations by Fetzer et al. (2020), we only included the countries in which more than 200 people participated, resulting in 47 countries and a sample of 93,125 respondents. In our sample, 44% was male, with an average age of 39.1 years (*SD* = 13.0) and average of 16.4 years of education (*SD* = 4.7). More details on the countries, the number of observations per day and respondents can be found in Table 1, Figure 1, and Table 2, respectively.

**TABLE 1:** Overview of the countries in the analysis.

**Country**	***N***	**%**
Argentina	886	0.95
Australia	930	1.00
Austria	1,067	1.15
Belgium	561	0.60
Brazil	11,564	12.42
Bulgaria	324	0.35
Chile	535	0.57
Colombia	1,628	1.75
Czechia	257	0.28
Denmark	500	0.54
Dominican Republic	543	0.58
Ecuador	291	0.31
France	2,715	2.91
Germany	10,096	10.84
Greece	325	0.35
Hungary	229	0.25
India	980	1.05
Indonesia	1,504	1.61
Israel	403	0.43
Italy	1,845	1.98
Japan	559	0.60
Kenya	377	0.40
Mexico	3,293	3.54
Morocco	377	0.40
Netherlands	1,416	1.52
New Zealand	351	0.38
Nigeria	213	0.23
Norway	296	0.32
Peru	1,151	1.24
Philippines	734	0.79
Poland	382	0.41
Portugal	546	0.59
Romania	793	0.85
Russia	3,366	3.61
Singapore	408	0.44
Slovakia	604	0.65
South Africa	542	0.58
South Korea	284	0.30
Spain	2,263	2.43
Sweden	5,852	6.28
Switzerland	4,184	4.49
Thailand	302	0.32
Turkey	2,850	3.06
Ukraine	1,441	1.55
United Kingdom	11,250	12.08
United States	11,423	12.26
Vietnam	685	0.74

**TABLE 2:** Means, standard deviations, and Pearson correlation matrix (*N* = 93,125).

**Variable**	***M***	***SD***	**1**	**2**	**3**	**4**	**5**	**6**	**7**	**8**	**9**	**10**	**11**	**12**	**13**	**14**	**15**	**16**	**17**	**18**
1. Measure stringency	0.69	0.16	–																	
2. Extraversion	4.16	1.58	−0.04***	–																
3. Measure stringency* Extraversion	2.89	1.28	0.49***	0.83***	–															
4. Depressive symptoms	1.72	0.64	0.06***	−0.13***	−0.08***	–														
5. Single or divorced^a^	1.44	0.50	0.08***	−0.10***	−0.04***	0.19***	–													
6. Number of household members	2.84	1.57	0.04***	0.02***	0.03***	0.00	−0.16***	–												
7. Monthly household income	4.12	1.38	0.03***	0.01*	0.02***	−0.08***	−0.19***	0.08***	–											
8. Participation at social gatherings^b^	8.93	23.08	−0.11***	0.05***	−0.03***	0.01	0.03***	0.02***	−0.04***	–										
9. Trust in government	2.83	1.49	0.03***	0.12***	0.11***	−0.19***	−0.10***	0.00	−0.08***	0.01***	–									
10. No health problems	0.87	0.34	−0.03***	0.00	−0.01***	−0.04***	0.01***	0.02***	0.02***	0.00	0.04***	–								
11. Neuroticism	3.35	1.45	0.06***	−0.14***	−0.08***	0.46***	0.13***	0.01***	−0.05***	0.00	−0.18***	−0.04***	–							
12. Openness	5.19	1.15	−0.02***	0.30***	0.24***	−0.08***	0.00	−0.03***	−0.01***	–0.01	0.01***	0.00	−0.16***	–						
13. Agreeableness	4.89	1.17	−0.09***	0.07***	–0.00	−0.11***	−0.03***	0.01	−0.03***	–0.00	0.11***	0.01*	−0.26***	0.14***	–					
14. Conscientiousness	5.19	1.27	–0.00	0.11***	0.10***	−0.24***	−0.12***	−0.01***	0.06***	−0.04***	0.10***	0.03***	−0.28***	0.12***	0.15***	–				
15. Day-to-day change COVID-19 cases	0.03	0.03	0.26***	0.06***	0.19***	−0.05***	−0.09***	−0.09***	−0.11***	−0.09***	0.26***	−0.01*	−0.10***	0.00	0.03***	0.06***	–			
16. Day-to-day change COVID-19 deaths	0.00	0.00	0.32***	0.03***	0.19***	−0.03***	−0.04***	−0.04***	−0.06***	−0.06***	0.14***	0.01*	−0.05***	−0.03***	0.03***	0.02***	0.71***	–		
17. Number of COVID-19 cases per capita	0.22	0.31	0.25***	0.07***	0.19***	−0.06***	−0.09***	−0.08***	−0.12***	−0.07***	0.30***	−0.02***	−0.11***	–0.01	0.03***	0.06***	0.90***	0.70***	–	
18. Number of COVID-19 deaths per capita	0.01	0.02	0.29***	0.01***	0.16***	−0.02***	−0.03***	−0.03***	−0.04***	−0.05***	0.12***	0.01**	−0.04***	−0.03***	0.02***	0.01***	0.59***	0.92***	0.68***	–

*p < 0.05, **p < 0.01, ***p < 0.001. M, mean; SD, standard deviation. ^*a*^0 = married/co-habiting, 1 = single/divorced. ^*b*^Frequency of participation in social gatherings.

### Measures

Descriptive statistics on all variables and a description of all variables and can be found in Tables 2, 3, respectively.

**TABLE 3:** Description of variables.

**Variable**	**Measure**	**Response categories**
Measure stringency	Included policy response measures are: - Workplace closing - Cancel public events - Restrictions on gathering size - Close public transport - Stay at home requirements - Restrictions on internal movement - Restrictions on international travel - Presence of public information campaigns	0 (no measures)–1 (total lockdown)
Depressive symptoms	How often have you been bothered by the following over the past 2 weeks? “Little interest or pleasure in doing things?” “Feeling down, depressed, or hopeless?” “Trouble falling or staying asleep, or sleeping too much?” “Feeling tired or having little energy?” “Poor appetite or overeating?” “Feeling bad about yourself—or that you are a failure or have let yourself or your family down?” “Trouble concentrating on things, such as reading the newspaper or watching television?” “Moving or speaking so slowly that other people could have noticed? Or so fidgety or restless that you have been moving a lot more than usual?”	1 (not at all)–4 (nearly every day)
Single or divorced	What is your marital status?	0 (married/co-habitating), 1 (single/divorced)
Number of household members	How many people live in your household?	
Monthly household income quintile	Country-specific income quintile to which the respondent belongs, based on the question: “What is your monthly household income, before tax, your country’s currency?”	1 (Lowest)–5 (Highest)
Participation at social gatherings	To what extent does the following statement describe your behavior for the past week? “I did not attend social gatherings.”	0 (does not apply at all)–1 (applies very much)
Trust in government	How much do you trust your country’s government to take care of its citizens?	1 (strongly distrust)–5 (strongly trust)
No health problems	Please consider the following list of health conditions: Cardiovascular diseases, diabetes, hepatitis B, chronic obstructive pulmonary disease, chronic kidney diseases, and cancer. How many of these conditions do you have?	0 (1 or more problems), 1 (no problems)
Personality traits Extraversion Neuroticism Openness Agreeableness Conscientiousness	I see myself as… “Extraverted, enthusiastic” and “Reserved, quiet” (reversed item) “Anxious, easily upset” and “Calm, emotionally stable” (reversed item) “Open to new experiences, complex” and “Conventional, uncreative” (reversed item) “Sympathetic, warm” and “Critical, quarrelsome” (reversed item) “Dependable, self-disciplined” and “Disorganized, careless” (reversed item)	1 (disagree strongly)–7 (agree strongly)
Day-to-day change COVID-19 cases	Day-to-day change in the total number of confirmed COVID-19 cases in the country of residence on the day the respondent participated based on John Hopkins COVID-19 data.	
Day-to-day change COVID-19 deaths	Day-to-day change in the total number of confirmed COVID-19 deaths in the country of residence on the day the respondent participated based on John Hopkins COVID-19 data.	
Number of COVID-19 cases	Total number of confirmed COVID-19 cases in the country of residence on the day the respondent participated based on John Hopkins COVID-19 data.	
Number of COVID-19 deaths	Total number of confirmed COVID-19 deaths in the country of residence on the day the respondent participated based on John Hopkins COVID-19 data.	
Age*	Which year were you born?	
Gender*	Which gender do you identify with?	0 (male), 1 (female)
Years of education*	How many years of education did you complete?	

*Used for the creation of the fixed effects.

#### Measure Stringency

The stringency of measures across country and time was measured using the COVID-19 Government Response Stringency Index (GRSI), that is up to date as of April 6, 2020 (Hale et al., 2020). The GRSI is comprised of sub-indexes on nine categories of protective measures: workplace closings, cancelation of public events, restrictions on gathering size, closing of public transport, stay at home requirements, restrictions on internal movement, restrictions on international travel and presence of public information campaigns. All sub-indexes differed in their scaling. For example, cancelation of public events had three categories: 0 (*no measure*), 1 (*recommend cancelling*) and 2 (*require cancelling*) and school closings had four categories: 0 (*no measures*), 1 (*recommended closing*), 2 (*require closing, only some levels or categories*) and 3 (*require closing, require closing all levels or categories*). Therefore, all sub-index scores were re-coded onto a 1–100 scale. These scores were then averaged into a single aggregate score ranging from 1 (*no measures*) to 100 (*total lockdown*). For interpretability purposes, Hale et al.’s (2020) original scale was recoded into a continuous scale from 0 to 1.

#### Extraversion

Extraversion was measured using the two-item measure from the Ten-Item Personality Inventory [TIPI; Spearman’s rho (ρ) = 0.53, Gosling et al., 2003]. The two items represent both poles of the extraversion dimension: “I see myself as extraverted, enthusiastic” and “I see myself as reserved, quiet.” Answer categories ranged from 1 (*disagree strongly*) to 7 (*agree strongly*). The measure was constructed by reverse coding the score on the “Reversed, quiet” item and computing an average score of the two items.

#### Depressive Symptoms

Depressive symptoms were measured using the average score respondents scored on the 8-item Personal Health Questionnaire (PHQ-8), e.g., “How often have you been bothered by the following over the past 2 weeks?…Little interest or pleasure in doing things” (α = 0.86, ω = 0.88, Kroenke et al., 2001; for validation in the general population, see Martin et al., 2006). Answer categories ranged from 1 (*not at all*) to 4 (*nearly every day*).

#### Covariates

We included several covariates that could confound the relationship between the stringency of measures, extraversion and depressive symptoms. In addition to typical demographic variables like age, gender, monthly household income, marital status and years of education, we also considered the 2-item TIPI measures of neuroticism, openness, conscientiousness and agreeableness (*ρ*s ranging from 0.18 to 0.52), trust in government, health problems, household composition, and participation in social gatherings over the past 5 days as covariates. At the country-day level, we controlled for the number of and day-to-day change in COVID-19 cases and the number of deaths per capita (see Dong et al., 2020).

### Analytical Strategy

To examine the moderating effect of extraversion on the relationship between measure stringency and depressive symptoms, we combine individual-level and country-level data and utilize a difference-in-difference analysis. Following Fetzer et al. (2020), we use the *reghdfe* package in Stata (Correia, 2016), which estimates linear regression models absorbing multiple levels (i.e., country-individual and time) of fixed effects. The advantage of a fixed model over a multilevel (random) effects model is that which takes out individual-specific heterogeneity (country-education and country-age-gender) at the country-level as well as (global) day-specific shocks.

We estimate the following regressions for all individuals from countries with at least 200 respondents who responded to the survey in the period March 20–April 6, 2020:

$$\begin{array}{c} \left(1\right) \end{array}\begin{array}{l} Depressive\;Symptoms_{ijt}=\;\\ {\text{β}}_{1}^{*}Measure\;stringency_{jt}+{\text{β}}_{2}^{*}Extraversion_{ijt}+\\ {\text{β}}_{3}^{*}Measure\;stringency_{jt}^{*}Extraversion_{ijt}+{\text{γ}}_{1}^{*}X_{ijt}+{\text{ γ}}_{2}^{*}X_{it}+\\ v_{j1}+v_{j2}+v_{t}+{\text{ε}}_{ijt}, \end{array}\;$$

where *DepressiveSymptoms*_*ijt*_ is depressive symptoms score of individual *i* in country *j* that responded to the survey on day *t*, *Extraversion*_*ijt*_ is an individual’s score on the extraversion index, and *MeasureStringency*_*jt*_ is degree of restrictions citizens have to face in country *j* on day *t. X*_*ijt*_ is a vector of individual-level control variables including income-level, marital status, comorbidities, and other personality characteristics, while *X*_*it*_ is a vector of country-level control variables including day-to-day change in COVID-19 cases and deaths per capita and the number of COVID-19 cases and the number of deaths per capita. In addition, we include country-education (*v*_*j1*_), country-age-gender (*v*_*j2*_) and day fixed effects (*v*_*t*_). Accordingly, we utilize the within-variation of people with certain characteristics that live within a particular country over time.

As some respondents filled out the questionnaire before strict measures were in place and others answered after countries’ lockdown, we can gauge to what extent changes in stringency measures differently affect extraverts and introverts’ mental health. In our estimations, standard errors are clustered by country-age and gender of the respondents. Weights were included to correct for socio-demographic differences between survey respondents and the general population in each country and differences in population size between countries (also see, Fetzer et al., 2020).

## Results

**FIGURE 2:**
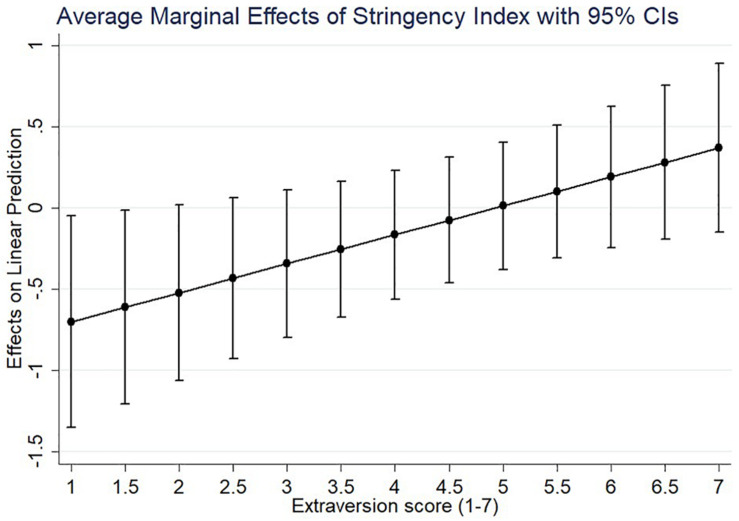
Visualization of extraversion moderating the relation between stringency measures and depressive symptoms. CI, confidence interval. Based on Estimations in Table 4, Model 3.

In line with our hypothesis and as exhibited in Table 4, extraversion moderated the relationship between measure stringency and depressive symptoms (β = 0.24, *p* < 0.05; Table 4, Model 2). Our conclusion holds when we control for individual-country and country-level control variables (β = 0.178, *p* < 0.05; Table 4, Model 3). Although extraversion is negatively related to depressive symptoms (β = −0.06, *p* < 0.01; Table 4, Model 1), for introverts, measure stringency has a negative effect on depressive symptoms, whereas, for extraverts, measure stringency has a positive, but not statistically significant effect depressive symptoms (see Figure 2). As an illustration, if the measure stringency index increases from 0 to 1, the depressive symptoms of extreme introverts decrease with 0.70 points (95% CI: −1.35 to −0.05), while they increase with 0.37 points for extreme extraverts (95% CI: −0.15 to 0.89). Model 3 in Table 4 also shows that being single or divorced, having health problems, having low trust in government, and having high degrees of neuroticism and conscientiousness (and to a lesser degree openness) are important correlates of reporting depressive symptoms in the early days of the pandemic.

**TABLE 4:** Results of regression analyses.

	**Model 1 coefficient (SE)**	**Model 2 coefficient (SE)**	**Model 3 coefficient (SE)**
Measure stringency (β_1_)	0.094 (0.275)	−0.763 (0.451)	−0.877 (0.394)*
Extraversion (β_2_)	−0.060 (0.010)***	−0.222 (0.063)***	−0.147 (0.050)**
Measure stringency*Extraversion (β_3_)		0.244 (0.099)*	0.178 (0.074)*
**Individual-level control variables**			
Single or divorced			0.090 (0.022)***
Number of household members			0.011 (0.012)
Monthly household income			−0.009 (0.007)
Participation in social gatherings over the past 5 days			−0.019 (0.051)
Trust in government,			−0.059 (0.008)***
No health problems			−0.142 (0.024)***
Neuroticism			0.189 (0.010)***
Openness			0.023 (0.010)*
Agreeableness			−0.004 (0.015)
Conscientiousness			−0.066 (0.011)***
**Country-level control variables**			
Day-to-day change COVID-19 cases			−0.469 (1.123)
Day-to-day change COVID-19 deaths			0.963 (12.85)
Number of COVID-19 cases per capita			−0.535 (0.175)**
Number of COVID-19 deaths per capita			1.415 (1.393)
Country by education fixed effects	Yes	Yes	Yes
Country-by age and gender fixed effects	Yes	Yes	Yes
Day fixed effects	Yes	Yes	Yes
Observations	93,125	93,125	93,125
Adjusted *r*-squared	0.62	0.63	0.71

*p < 0.05, **p < 0.01, ***p < 0.001. β, unstandardized regression coefficient; SE, standard error.

## Discussion

Our findings provide support for the hypothesis that extraversion moderates the relationship between stringent COVID-19 protective measures and depressive symptoms. The assumption that the stringent measures are beneficial for introverts and detrimental for extraverts received only partial support. The results indicated that introverts indeed fare substantially better when living in a country that has installed stringent protective measures than in countries that did not. However, even though the lifestyle associated with social distancing seems to feel more unnatural to extraverts than to introverts, the damaging effect of living in a country where the government imposed stringent measures appears to be limited for extraverts. This conclusion is underscored by the negative association between extraversion and depressive symptoms in our sample. Indeed, psychological research indicates that, compared to introverts, extraverts are less susceptible for mental illnesses (Malouff et al., 2005), such as depression and anxiety (Spinhoven et al., 2014), and generally happier (Steel et al., 2008; Anglim et al., 2020).

### Limitations and Future Research

These findings should be interpreted within the limitations of this research. First, we were not able to empirically test our assumption that it is the negative social consequences of stringent measures that explain the slight increase of depressive symptoms among extraverts. We, however, believe that this is the most plausible explanatory mechanism, as research suggests that it is the loss in social connection that causes extraverts to suffer more mentally during this pandemic (Folk et al., 2020).

Second, even though our analytical strategy allowed us to take out individual-specific heterogeneity, and extraversion is a relatively stable personality trait (Damian et al., 2019), the cross-sectional nature of the survey data, collected in the early days of the pandemic, did not allow us to examine whether introverts’ and extraverts’ responses to the protective measures changed as the situation evolved. Since the period of data collection, time has not stood still. For example, after the first wave of infections was contained, countries started relaxing protective measures. A while later, many of these countries again imposed protective measures to prevent a second wave of infections to crop up. Testing our hypothesis on more recent data is an important direction for future research for, at least, two reasons. Research on well-being set-points and coping in times of crisis suggests that people have the tendency to adapt to adversity as a crisis evolves (Riolli et al., 2002; Cummins and Wooden, 2014). If, in time, extraverts find new ways to satisfy their need for social connections (e.g., virtual communication), the interaction effect could disappear. Furthermore, the charm of social distancing for introverts may be only temporal, because, if social distancing becomes the new normal, introverts may struggle with getting sufficient social support (Blue, 2020).

Third, with a broader set of measures, we would have been able to draw more robust conclusions. As extraversion is a multi-facetted construct (Soto and John, 2017) and not all facets contribute to mental health in equal degrees (Margolis et al., 2020), it could be that measure stringency only significantly interacts with one or two facets of extraversion. In a similar vein, it could be that the moderating effect of extraversion effects would have be more apparent for more fluctuant mental health constructs, such as daily positive and negative affect (Hudson et al., 2017). In addition, type of house and living situation could be interesting variables to consider, as people living in a more spacious house or more rural areas might have had more opportunity to organize social gatherings at a safe distance and maintain a high degree of personal space vis-à-vis other household members and, in turn, suffered less from the social consequences of the pandemic. Researching the role of daily time use would be a worthwhile endeavor too, as research conducted during the early days of COVID-19 shows that activities vary drastically in the extent to which they make people happy (Lades et al., 2020). Finally, we believe that studying the role of internet availability and familiarity with virtual communication media could be a fruitful research direction, as these factors could be essential for people to maintain social contacts when facing stringent measures.

Fourth, the surveying procedure may have influenced the external validity of our findings. First, Fetzer et al.’s (2020) snowballing procedure may have resulted in certain populations to be overrepresented (e.g., women) or underrepresented in our sample (e.g., individuals in lower social strata). Even though weights were used to correct for socio-demographic differences between survey respondents and the general population in each country, still some groups might be completely absent. Most notably, by administrating a web-based survey, Fetzer et al. (2020) excluded individuals that do not have access to the internet (e.g., underprivileged people) or lack the knowledge to use it (e.g., elderly people, Baltar and Brunet, 2012). It is perhaps this overlooked proportion of the population that may have been most negatively affected by the social consequences of the pandemic, as it had limited opportunity to maintain social relationships when physical contact was infeasible. Therefore, we recommend researchers to use data based on probability sampling methods and a variety of survey modes (e.g., paper or telephone survey) when replicating our study in future research.

## Conclusion

All in all, our results provide empirical evidence on a popular, but mostly unsubstantiated assumption that extraverts suffer more from COVID-19 protective measures than introverts. Nevertheless, as, in the end, extraverts and introverts both have an innate need for human connection (Baumeister and Leary, 1995), it will be essential to develop and test interventions that help people to cope with the pandemic’s social consequences (Steele, 2020). It may, for instance, be worthwhile to develop public information programs that incentive citizens to adhere the COVID-19 protective measures and, at the same time, to help people maintain social relationships and stay mentally fit, e.g., combining outdoor activities with social interaction (Lades et al., 2020) and making responsible use of virtual communication tools to stay in touch (Garfin, 2020).

## Data Availability Statement

The data and code used for this study can be found at https://osf.io/vgkmd/. The original data from Fetzer et al. (2020) can be found at https://osf.io/3sn2k/.

## Ethics Statement

The data collection procedure was reviewed and approved by the Massachusetts Institute of Technology (reference: E-206, see Fetzer et al., 2020). The patients/participants provided their written informed consent to participate in this study.

## Author Contributions

IW wrote most of the manuscript and verified the results. SS wrote a part of the manuscript and verified the results. MB ran most of the analyses and came up with the research question. All authors contributed to the article and approved the submitted version.

## Conflict of Interest

The authors declare that the research was conducted in the absence of any commercial or financial relationships that could be construed as a potential conflict of interest.
